# Expansion of a Population of Large Monocytes (Atypical Monocytes) in Peripheral Blood of Patients with Acute Exacerbations of Chronic Obstructive Pulmonary Diseases

**DOI:** 10.1155/2018/9031452

**Published:** 2018-05-17

**Authors:** Jing Yang, Man Qiao, Yanxia Li, Guohua Hu, Chunxia Song, Liping Xue, Hong Bai, Jie Yang, Xi Yang

**Affiliations:** ^1^Department of Immunology, Tianjin Key Laboratory of Cellular and Molecular Immunology and Key Laboratory of Educational Ministry of China, School of Basic Medical Sciences, Tianjin Medical University, Tianjin, China; ^2^Department of Pharmacology, Institute of Acute Abdominal Diseases, Tianjin Nankai Hospital, Tianjin, China; ^3^Department of Respiration, Tianjin Nankai Hospital, Tianjin, China; ^4^Department of Immunology, Max Rady College of Medicine, University of Manitoba, Winnipeg, MB, Canada

## Abstract

Acute exacerbation of chronic obstructive pulmonary disease (AECOPD) is closely associated with airway inflammation including monocytes, lymphocytes, and neutrophils. Monocytes play an essential role in the pathogenesis of chronic obstructive pulmonary disease (COPD). To elucidate the association of circulating monocyte alteration with AECOPD, we analyzed monocyte subpopulation in the peripheral blood of 16 healthy volunteers and 22 AECOPD patients at the stages of admission and remission after clinical therapy. We found a dramatic increase of a previously unreported population of large size circulating atypical monocytes (A Mo) in AECOPD patients, characterized by higher forward scatter and lower side scatter values than the typical monocytes (T Mo) which were observed predominantly in healthy individuals. Further analysis showed that A Mo expressed higher levels of CD16, intercellular adhesion molecule 1 (ICAM-1), and chemotactic protein-1 receptor-2 (CCR2) than T Mo. In contrast, the expression of class II antigen (HLA-DR) by A Mo was lower than T Mo. More importantly, we observed that the percentage of circulating A Mo among total monocytes correlated with the length of hospital stay (time to remission) and disease duration. The data suggest that circulating A Mo might have the potential to serve as a biomarker in the diagnosis and prognosis of AECOPD.

## 1. Introduction

COPD is defined as a progressive and irreversible decline in lung function caused by airflow obstruction which is associated with chronic airway inflammation in response to cigarette smoke or other noxious particles. The chronic inflammation often persists despite smoking cessation. Altered innate immune cell function appears to play a key role in the development and progression of COPD [1, 2]. On the one hand, increased numbers of monocytes, macrophages, and neutrophils traffic prominently into a patient airway; on the other hand, these invasive leukocytes exhibit defective function resulting in chronic bacterial colonization and perpetual infection. The dysregulation of the innate immune system in COPD is even more serious in the status of acute exacerbation [3].

AECOPD could have significant detrimental effects on patients, leading to the loss of lung function [4] and a reduced quality of life [5, 6] with a poorer survival rate [7]. The exacerbations are often caused by viral and/or bacterial infections in the respiratory tract accompanied by extravagant inflammatory responses [8, 9]. The influx of leukocytes in the lung of COPD/AECOPD patients is comprised of a variety of cells, including lymphocytes, neutrophils, and cells from the mononuclear phagocytic system (MPS), including monocytes, macrophages, and DCs [10]. Macrophages are professional antigen-presenting cells and are specialized to maintain airway sterility. However, monocytes/macrophages in COPD patients, although increasing in number, show reduction in the phagocytic and killing function for clearing infectious agents and apoptotic cells [11–13], resulting in persistent bacterial colonization, necrotic material accumulation, and subsequent perpetuation of inflammation.

Accumulated macrophages in the airway originate from both tissue-resident macrophages and alveolar macrophages and from monocyte-derived macrophages recruited from the circulation [14, 15]. Blocking CC chemokine receptor 2 (CCR2), a critical regulator of monocyte trafficking, reduced monocyte accumulation and neutrophil influx in the airway in a mouse model [16], suggesting that newly recruited monocytes might participate in the inflammatory process. Similarly, the depletion of monocytes but not neutrophils could prevent lung emphysema induced by cigarette smoke exposure in rats [17]. The relationship between the circulating monocytes and airway inflammation raises the question whether it is possible to predict the inflammation in the lung by studying peripheral blood. Recent studies have established the heterogeneity and plasticity of circulating monocytes in animals and humans [18, 19]. Data from several groups also indicated that the varying patterns of monocyte subsets were associated with disease progression or prognosis [20–22]. However, it remains unclear for the changes in macrophage subpopulations especially circulating monocytes in a relationship with COPD or AECOPD.

In the present study, we evaluated the number, size, granularity, and surface markers of circulating monocytes in the peripheral blood of AECOPD patients in comparison with healthy controls. Our data show a dramatic increase of a novel A Mo population of monocytes in AECOPD patients. This novel A Mo population is characterized by a larger size and higher expression of surface CD16, ICAM, and CCR2 markers than T Mo in the healthy controls. About 60% of the monocytes in AECOPD patients were A Mo while A Mo was nearly negligible in the healthy controls. In addition, the A Mo population appeared expressing lower levels of HLA-DR, suggesting alteration in immune function. More importantly, we found that the percentage of A Mo in AECOPD patients correlated with hospital stay and disease duration of the patients. The finding suggests a novel classification strategy for monocyte subsets in the blood of AECOPD patients and provides the first proposal regarding the presence and definition of atypical monocytes, which provides new insights into the underlying roles of monocytes in the pathogenesis and prognosis of AECOPD. It also suggests that A Mo in circulation might have the potential to be a biomarker for the diagnosis and prognosis of COPD/AECOPD.

## 2. Materials and Methods

### 2.1. Study Subjects

This study population consisted of 16 healthy never-smoking donors (H), 22 hospitalized AECOPD patients at admission (Admission) and 17 AECOPD patients at remission (Remission) in Tianjin Nankai Hospital. All subjects provided fully informed consent in this study.

Cases of COPD were defined according to the Global Initiative for Chronic Obstructive Lung Disease (GOLD) guidelines [23]. AECOPD was diagnosed as acute altered respiratory symptoms requiring additional treatments including oxygen, antibiotics, or systemic corticosteroids to meet the Anthonisen diagnostic criteria [24]. Lung function and chest radiography results were obtained for all participants. None of the patients had other serious diseases, such as asthma, allergic rhinitis, tuberculosis, or cancer. General data including age, gender, routine blood tests, and serum C-reactive protein (CRP) levels were recorded (Table 1).

### 2.2. Sample Preparation and Flow Cytometry Analysis

Peripheral venous blood samples were collected from healthy donors and AECOPD patients. Flow cytometric analysis of monocyte subsets was performed according to the forward scatter (FSC) and side scatter (SSC) as well as surface marker expression. Briefly, 100 ml whole blood was collected from each individual into an anticoagulant-coated tube (EDTA). Subsequently, the following antibodies were added into the blood sample and incubated for 15 minutes at room temperature in the dark: anti-CD14-PercP-Cy™5.5 (clone M*φ*P9), anti-CD16-FITC (clone 3G8), and anti-CD54-APC (ICAM-1) (clone HA58), and Ig-matched isotypes were all purchased from BD Pharmingen; anti-HLA-DR-PE (clone LN3) and the corresponding isotype were purchased from eBioscience; anti-CCR2-APC (clone K036C2) and the corresponding isotypes were purchased from BioLegend. Then, 1 ml of lysis solution (BD Biosciences) was added to lyse erythrocytes. Finally, harvested cells were washed and analyzed with flow cytometry immediately. The four-color analysis was performed, and the expression levels of the surface markers mentioned above were measured on a FACSCalibur flow cytometer (BD Biosciences) equipped with 488 nm blue and 633 nm red lasers and analyzed by FlowJo software (Tree Star Inc., Ashland, OR). Surface molecule levels were expressed as the percentages and mean fluorescence intensity (MFI) values. A minimum of 100,000 events of the total cells was acquired.

### 2.3. Collection and Analysis of Laboratory and Inflammatory Parameters

Venous blood samples were collected and centrifuged for 10 min at 3000 rpm/min at 4°C. Serum CRP levels were determined by an ADVIA2400 Chemistry System (Siemens AG, Germany) according to the manufacturer's instructions. A routine test of blood leukocytes was done using the Sysmex XE-2100-automated blood cell counter (Sysmex, Kobe, Japan). Serum cytokine levels of IL-6 and IL-8 were measured by a Cytometric Bead Array kit (CBA, BD Pharmingen) according to the instructions. Briefly, serum samples (50 *μ*L) were inoculated with 50 *μ*L of capture microbeads and 50 *μ*L of PE-conjugated detection reagents (anti-human IL-6 and anti-human IL-8) in the dark at room temperature. Three hours later, samples were washed and collected. Data were analyzed using the BD FCAP Array Software version 1.0.1 (BD Biosciences). All AECOPD patients underwent chest radiography to characterize the nature of their lung disease.

### 2.4. Statistical Analysis

The data are represented as the mean ± standard deviation (SD). For comparing distributions between groups, the nonparametric Newman-Keuls test was used, and for two independent groups, Student's *t*-test was performed. A *P* value less than 0.05 was considered to be statistically significant. A linear regression analysis was performed to examine the relationship between atypical monocyte count and total monocytes. All initial statistical calculations were done with GraphPad Prism 5 (GraphPad Software Inc., La Jolla, CA).

## 3. Results

### 3.1. Patients with AECOPD Exhibit Systemic Inflammation

Clinical features, spirometric data, and laboratory parameters of AECOPD patients and healthy controls are detailed in Table 1. Compared to the values in healthy controls, the forced expiratory volume in one second/forced vital capacity (FEV1/FVC) and forced expiration volume in one second % predicted FEV1 (FEV1% predicted) values were dramatically lower in patients with AECOPD (*P* < 0.001). All patients were given routine blood examination twice, once at admission (AECOPD) and again at remission (Remission). The results showed that at admission, the absolute numbers of white blood cells (WBCs), neutrophils, and monocytes, as well as the percentages of neutrophils, were significantly increased in AECOPD patients compared with healthy controls, while the percentage of lymphocytes was lower in patients compared to controls (*P* < 0.01). Additionally, serum C-reactive protein (CRP) and IL-6 and IL-8 levels were also significantly higher in AECOPD patients than in the control group. At remission, the increased neutrophil count and percentage of neutrophils observed in AECOPD patients were decreased significantly compared to the values at admission (neutrophil count: 5.77 ± 2.14 versus 7.46 ± 3.27, resp., *P* < 0.05; neutrophil %: 68.00 ± 9.94 versus 76.46 ± 14.44, resp., *P* < 0.05). In contrast, the previously decreased lymphocyte percentage was elevated at remission compared to at admission (lymphocyte %: 21.65 ± 8.19 versus 15.92 ± 10.93, resp., *P* < 0.05), although it had still not returned to normal levels. Notably, although the number of monocytes was significantly increased in AECOPD patients compared with healthy controls (*P* < 0.01), there was no marked decline in monocyte numbers at remission. Additionally, the serum CRP level was reduced nearly five folds at remission compared to at admission (9.94 ± 7.91 versus 39.93 ± 28.89, resp., *P* < 0.01).

### 3.2. Atypical Monocytes Expand Dramatically in Patients with AECOPD

**Figure 1:**
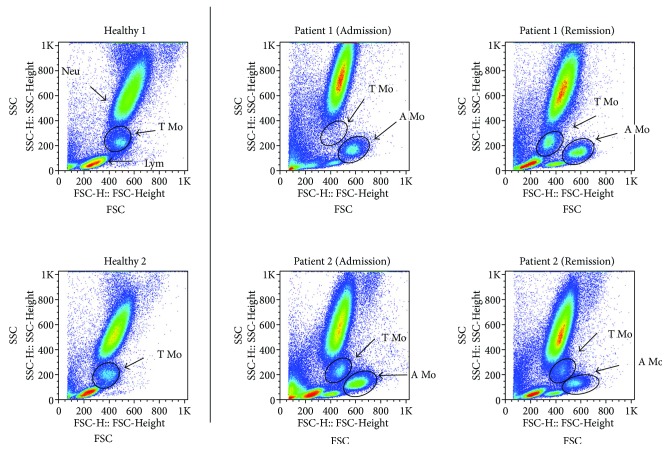
Gating strategy for the characterization of monocyte population. Representative dot plot of the whole blood cells from normal healthy donors and patients with AECOPD at admission by flow cytometry. Mo: monocytes; Neu: neutrophils; Lym: lymphocytes.

**Figure 2:**
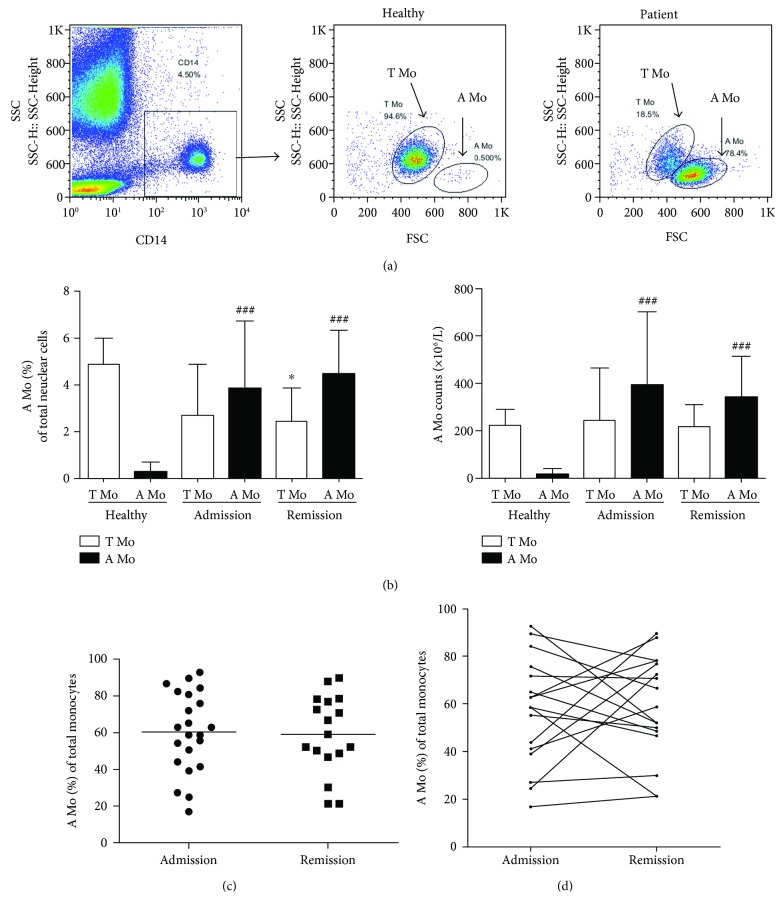
Light scatter distribution for T Mo and A Mo of healthy donors and AECOPD patients at admission and remission. (a) T Mo and A Mo in the CD14^+^ population of AECOPD patients. (b) Summary of monocyte subsets in AECOPD patients and control subjects. The percentage (left) and absolute number (right) of T Mo (open bar) and A Mo (black bar) monocyte subset from the healthy control group, and patients at admission and remission are shown as the mean ± SD. Comparisons were performed by ANOVA with the nonparametric Newman-Keuls test. ^∗^*P* < 0.05, compared with T Mo of healthy individuals. ^###^*P* < 0.001, compared with A Mo of healthy subjects. (c) Frequency of A Mo in total CD14^+^ monocytes from AECOPD patients (*n* = 17) at admission and remission. (d) Comparison of the percentage of A Mo in total CD14^+^ monocytes from each AECOPD patient at admission and remission. The results were analyzed by Student's *t*-test.

We further focused on the monocytes in AECOPD patients at admission and at remission after clinical therapy. As shown in Figure 1, scatter profiling clearly distinguished monocytes from bigger neutrophils (Neu) and smaller lymphocytes (Lym) by flow cytometry. Unlike the healthy controls that showed a rather universal monocyte population (T Mo), the monocytes in AECOPD patients were divided into two subpopulations of different sizes. The larger one with higher forward scatter (FSC) values was designated as “atypical monocytes or A Mo,” and the smaller one which was similar as those in healthy controls was designated as “typical monocytes or T Mo.” When we gated CD14^+^ cells (monocytes) for scatter profiling, it also showed the separate A Mo population in AECOPD patients which was not seen in healthy controls (Figure 2(a)). We also examined the percentages and absolute numbers of these subpopulations (T Mo and A Mo) in AECOPD patients before (Admission) and after (Remission) successful clinical therapy in comparison with healthy subjects. As summarized in Figure 2(b), about 60% of monocytes in AECOPD patients were A Mo while virtually all the monocyte healthy controls were T Mo. When the absolute number of monocyte subsets was examined, it was found that the T Mo population in AECOPD was similar to that in healthy controls, so the difference of monocytes between the patient and the control was mainly in the A Mo population. The results suggest that expansion of A Mo is a characteristic change in the blood of AECOPD patients. Furthermore, although the patients at remission have achieved clinical improvement, the percentage of A Mo among total monocytes, on average, had no significant changes (Figure 2(c)). Although some individuals had changes in the percentage of Mo at remission, the trend was not consistent, namely, both increases and decreases being found (Figure 2(d)).

### 3.3. Differential Surface Marker Expression by A Mo and T Mo

**Figure 3:**
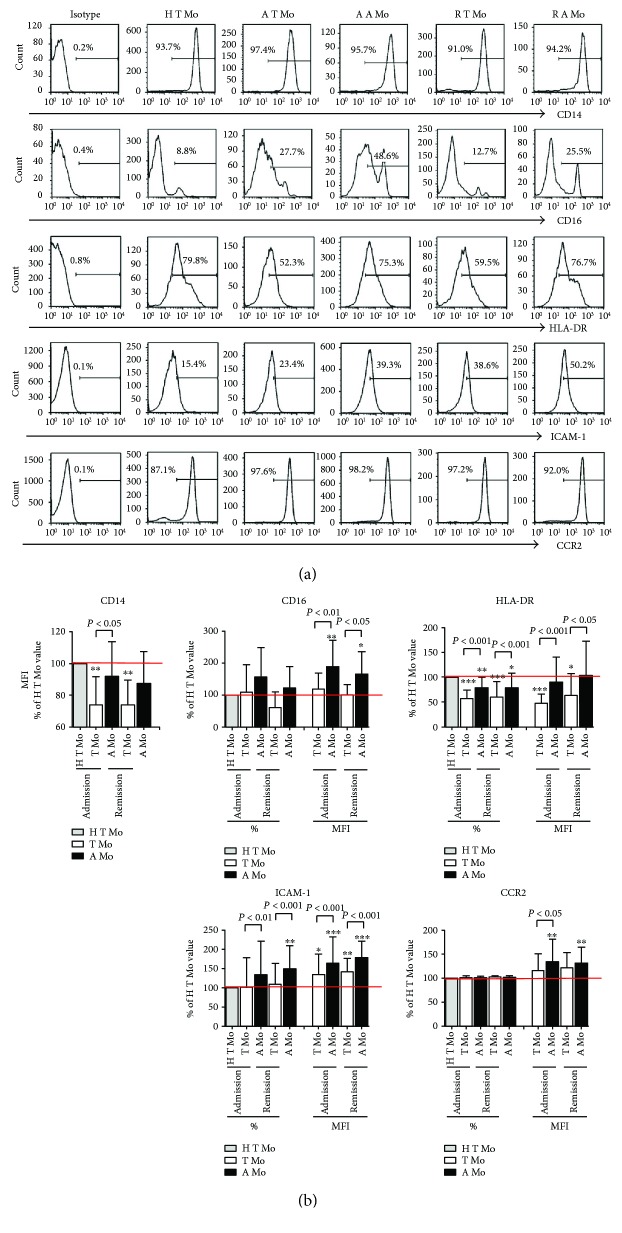
Surface phenotype of T Mo and A Mo from AECOPD patients at admission (*n* = 22) and at remission (*n* = 17) as well as from the control group (*n* = 16). (a) Representative shows altered expression of surface markers of CD14/CD16/HLA-DR/ICAM-1/CCR2 in T Mo of healthy donors (H T Mo), from AECOPD patients at admission (A T Mo) and remission (R T Mo), as well as in A Mo of AECOPD patients at admission (A A Mo) and remission (R A Mo). (b) Mean data of the percentage and MFI of surface marker expression of T Mo (white bar) and A Mo (black bar) from patients at admission and at remission are represented. Values are expressed as the mean ± SD, and Student's *t*-test and the nonparametric Newman-Keuls test were used for comparisons between groups. ^∗^*P* < 0.05, ^∗∗^*P* < 0.01, and ^∗∗∗^*P* < 0.001, compared with T Mo of healthy subjects.

To unravel the difference in the phenotypic signature between T Mo and A Mo, we compared the levels of CD14, CD16, ICAM-1, CCR2, and HLA-DR on these two subpopulations in patients and controls (Figure 3(a), Table 2). The expression levels of these molecules on the typical monocytes of healthy controls (H T Mo) were taken as baselines to which the levels on T Mo and A Mo of AECOPD patients at admission and at remission were compared (Figure 3(b)).

The data showed that the density (MFI) of CD14 was lower on T Mo and A Mo of AECOPD patients compared with monocytes (T Mo) from the healthy group (*P* < 0.01). The expression level of CD16 (% and MFI) increased dramatically on A Mo but not on T Mo of patients with AECOPD compared with those from healthy controls.

Reduced expression of HLA-DR molecules on monocytes is associated with a depressed immune status, especially in critically ill patients [25]. We found that both the percentage and the MFI of HLA-DR on T Mo were significantly lower in AECOPD patients than in healthy donors. Similar results could be observed for A Mo, but to a relatively lower degree. Increased ICAM-1 levels (MFI) on both subpopulations were found in patients compared with controls, and this upregulation was more pronounced on A Mo than on T Mo (*P* < 0.001).

CCR2 is an important chemokine receptor that is involved in the chemotaxis of specific monocyte subsets [26, 27]. We observed that only A Mo expressed higher levels of CCR2 than T Mo from healthy controls.

Notably, there were no significant differences in the expression of CD14, CD16, ICAM-1, HLA-DR, and CCR2 on any subset when cells from the same patient were compared before (at admission) and after clinical treatment (at remission).

Taking together, the phenotypic analysis showed increased expression of CD16, ICAM-1, and CCR-2, but decreased CD14 (density) and HLR-DR on A Mo.

### 3.4. Correlation of A Mo Amounts (Percentage and Absolute Number) with Disease Duration

**Figure 4:**
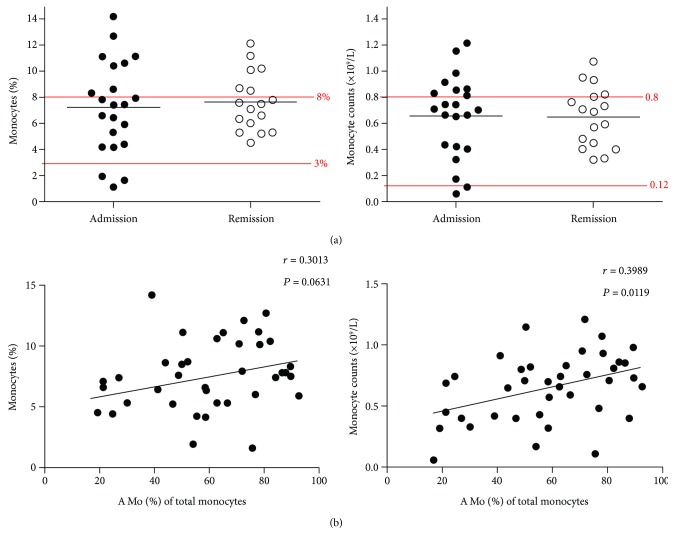
Relationships between A Mo and total monocytes. (a) Frequency (left panel) and absolute number (right panel) of circulating CD14+ monocytes in AECOPD patients at admission (*n* = 17) and remission (*n* = 17). The normal range of the percentage and the absolute number of monocytes were labeled in red line. (b) Correlation analyses of the proportion of A Mo with CD14+ monocytes in the peripheral blood of patients with AECOPD at admission (*n* = 22) and remission (*n* = 17). Each dot represents one individual. Simple linear regressions are shown.

To study the relationship between circulating monocytes and the disease status, we examined total and A Mo monocytes with several parameters. First, we examined the total monocyte (CD14^+^) levels of AECOPD patients at admission and remission. As shown in Table 1 and Figure 4(a), there was no significant difference in the frequency and absolute number of CD14^+^ total monocytes in AECOPD patients before and after clinical treatment. Not surprisingly, a simple linear regression analysis revealed that there was a trend for a positive correlation between the percentage of A Mo among total monocytes and the frequency of total monocytes among blood nuclear cells (*P* = 0.0631). There was additionally a strong positive correlation between the percentage of A Mo among monocytes and the absolute number of monocytes (*P* = 0.0119, Figure 4(b)). The results confirm the finding in Figure 2(b) that the predominant increase of monocytes in AECOPD patients was the A Mo subpopulation.

**Figure 5:**
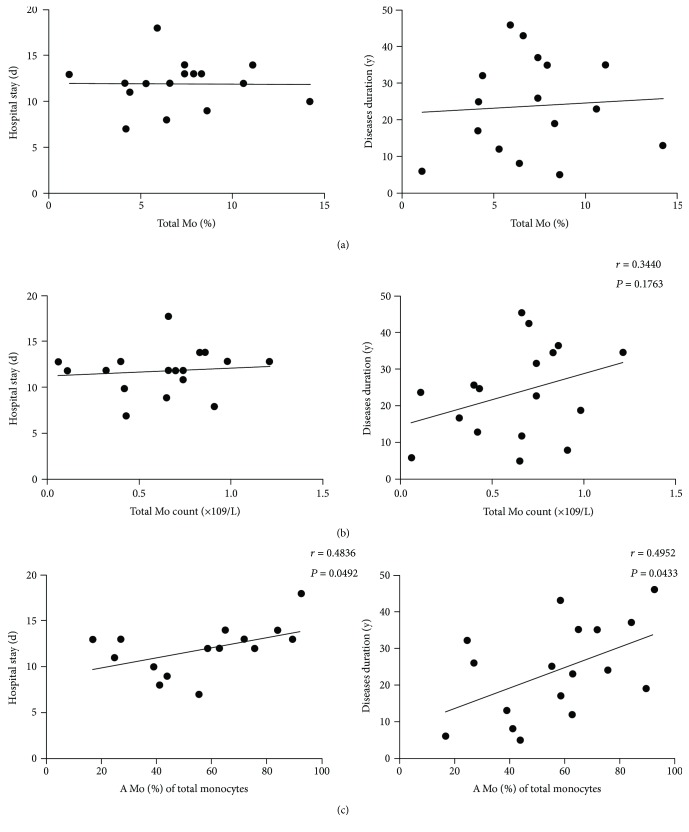
Correlations between numbers of A Mo and clinical parameters of patients with AECOPD. Relationships between the percentage of total monocytes (a) and total monocyte counts (b) and the percentage of A Mo in total monocytes (c) with the length of stay in the hospital of current acute exacerbate of COPD patients and duration of COPD (*n* = 17). Each dot represents one individual. Simple linear regressions are shown.

We further examined the relationship of total monocyte and A Mo with patient hospital stay and disease duration. Interestingly, we found that the proportion of A Mo among total CD14^+^ monocytes, but not the proportion of total monocytes or the monocyte count, among the blood nuclear cells correlated positively with the length of hospital stay (*r* = 0.4836, *P* = 0.0492) and the length of disease duration (*r* = 0.4952, *P* = 0.0433). The results suggest that A Mo proportion might be a better marker for predicting AECOPD outcomes, particularly hospital stay and disease duration (Figure 5).

## 4. Discussion

In this study, for the first time, we demonstrated a high count of a population of large size monocytes, termed A Mo in the blood of AECOPD patients. These cells can be distinguished from the smaller typical monocytes that prevail in healthy individuals. About 60% of the monocytes in AECOPD patients were A Mo, which was negligible in healthy controls. In comparison with T Mo, A Mo exhibited higher levels of surface marker expression (CD14, CD16, HLA-DR, ICAM-1, and CCR2) in AECOPD patients, suggesting higher biological activity of A Mo. More importantly, we found that the proportion of A Mo showed a significantly positive correlation with the length of hospital stay and the years (duration) of the COPD history of AECOPD patients, which suggested that A Mo might be a predictive parameter for disease assessment of AECOPD in diagnosis and prognosis, at least for short-term outcomes.

In the 1980s, after monocytes were purified by density gradient, several groups found that human monocytes were comprised of two populations that differed in size and density [28–30]. Recently, based on the recognition of surface antigens by CD14 and CD16 monoclonal antibodies, three monocyte subsets were identified: a CD14^++^CD16^−^ classical subset, a CD14^++^CD16^+^ intermediate subset, and a CD14^+^CD16^++^ nonclassical subset [31]. Using two-color fluorescence and morphological analysis, Ziegler et al. found that the three subsets differed in size: CD14^++^CD16^−^ > CD14^++^CD16^+^ > CD14^+^CD16^++^ [32]. Another more recent study of an animal model also demonstrated that the Ly-6C^high^ subset was larger than the Ly-6C^low^ subset with higher FSC/SSC values [33]. Correspondingly, our study confirmed the inhomogeneous size of monocytes. In addition, we found that A Mo and T Mo in AECOPD patients were different not only in physical properties (size and granularity) but also in surface marker expression.

CD14, CD16, and CCR2 are the best-known surface markers for circulating monocyte identification. The proportion of CD16^+^ monocytes is less than 5% in healthy individuals but rises with age [34], during infections [35, 36], and in patients with coronary artery disease [37] or periodontitis [38]. The interaction of chemokine receptor CCR2 with its ligand chemoattractant protein-1 (MCP-1, CCL2) is responsible for the migration of monocytes from circulation into a local inflammatory site [39–41]. ICAM-1 is constitutively expressed at low levels on the surface of epithelial cells, endothelial cells, and human monocytes under normal conditions but is increased during infection and inflammation, one of the primary functions of which is to regulate leucocyte infiltration and migration during respiratory infections [42, 43]. In this study, we found that the expression of CD16, CCR2, and ICAM-1 increased significantly on the surface of A Mo in patients with AECOPD compared with healthy controls, which implied that A Mo but not T Mo from AECOPD patients might exhibit stronger migratory ability. Therefore, we postulate that in contrast to the smaller T Mo that circulate in the peripheral blood, A Mo might contribute to the mobilization and migration of monocytes into local lung tissues in AECOPD. In fact, there are some clues to support our hypothesis. For example, a group described the percentage of a population of small macrophages that was significantly increased in induced sputum of COPD patients; these cells were considered monocyte/macrophage lineage cells based on the presence of CD14 and HLA-DR antigens [44]. This was consistent with the study by Rosseau et al*.,* who reported that alveolar cells in acute respiratory distress syndrome (ARDS) patients were newly settled from the blood and shared a phenotype with circulating monocytes [45]. Correspondingly, several groups have demonstrated higher MCP-1 concentrations in sputum, plasma, and bronchoalveolar lavage (BAL) of both acute exacerbation and stable stage COPD patients compared to healthy controls [46, 47], and MCP-1 and CCR2 polymorphisms are considered new risk factors for COPD [48].

The expression of HLA-DR on monocytes/macrophages as measured by flow cytometry has been considered to be an indicator for predicting the occurrence of infections and to be related with outcomes [49]. Therefore, it is likely that monocytes, both T Mo and A Mo, are defective in AECOPD patients, and decreased HLA-DR level might contribute, at least in part, to the immunosuppressed or immune tolerant status of COPD patients [50] and even result in the frequent occurrence of AECOPD.

Considering the positive correlation between the percentage of A Mo and the total CD14^+^ monocytes in circulation, our data indicate the possibility that increased total monocytes in the blood of AECOPD patients might be due to the occurrence of atypical monocytes. A question is then raised: where do atypical monocytes originate from? Are they released directly from the bone marrow or from other reservoirs such as the spleen, or do they differentiate from circulating monocytes in the blood? We cannot yet propose a conclusive answer to this question in the present research. It is well known that monocytes do not proliferate. In an inflammatory condition, the influx of monocytes in the bloodstream and lung might likely depend on mobilization from the bone marrow mediated by some critical molecules, such as CCR2 [39–41, 51]. Additionally, there has been a general agreement that monocytes develop from the haematopoietic stem and progenitor cells in the bone marrow via several sequential steps [52, 53]. Compared with mature monocyte in circulation, immature precursors such as monoblasts and promonocytes, which could not be observed in the peripheral blood of healthy individuals, are larger and exhibit lower granularity [54, 55], which seemed in line with the features of A Mo in our study. Notably, after clinical management, neither the increased number nor the altered expression levels of surface molecules on A Mo were observed to recover. These data might possibly explain why COPD patients with a history of AECOPD seem more inclined to frequently experience exacerbations [56].

However, there are no effective biomarkers to distinguish the diagnosis and outcome predictions of COPD/AECOPD. Molecular changes and gene expression profiling in the lung tissue might directly reflect lung pathology in the progression of COPD; however, lung tissue samples are not routinely accessible [57]. Hence, until now, the best predictor of exacerbations in stable COPD has been based on previous exacerbation events [58]. Whether inflammatory biomarkers can be applied in evaluating or predicting exacerbation events or outcomes in AECOPD patients is still a topic of debate. Previous studies have determined that some inflammatory biomarkers such as CRP, inflammatory cells, and fibrinogen are associated with poor outcomes and an increased onset risk of exacerbations in patients with AECOPD [59–61]. In contrast, other studies have found that these biomarkers are helpful in clinical practice but are far from ideal in AECOPD assessments and prognoses, because most of them are nonspecific. However, the combination of several inflammatory biomarkers is still important and has been recommended [58]. Unlike the other two inflammatory immune cells that are predominant in the circulation, neutrophils that are related to innate immune response to bacterial infection and lymphocytes related to adaptive immune responses against the detailed biological effects of monocytes remain relatively ambiguous [62]. Additionally, there is still controversy regarding whether monocytes can be used as a valuable clinical indicator. Similarly, we also found that the numbers of total monocytes varied in AECOPD patients, but dramatically elevated A Mo numbers could be seen in nearly all the patients enrolled in this study. More importantly, A Mo but not total monocytes were positively associated with short-term outcomes (the length of hospital stay and disease duration) in AECOPD patients. A Mo might be a more sensitive and specific biomarker than total monocytes.

Although the finding of the predominant expansion of A Mo as a potential biomarker in predicting AECOPD hospital stay is encouraging, much more study is needed before it becomes a reality in clinical use. One of the major limitations of the study is the relatively small size of the samples, especially the control group, which were from healthy donors of younger ages than the patients. Second, although the phenotypic changes have provided some clue of the functional difference of A Mo from T Mo of healthy individuals and patients, there was no experimental study to confirm this. Thirdly, further characterization including the transcriptome study of the novel A Mo population is needed for its origin, function, migration, and distribution. Notably, we found that there are no significant correlations between the percentage of A Mo among monocytes with the systemic inflammatory parameters in the stages of admission and remission. As shown in Table 1, many of the inflammation parameters were improved at remission, but the A Mo population in average had no significant changes including their tested surface markers. The phenomena in one way might suggest the lack of correlation between A Mo with systemic inflammation but in the other way might be because of the smaller sample size of the study. Indeed, nearly half of the patients showed a reduction of A Mo at remission compared to admission (Figure 2(d)), which might be more consistent when large samples are tested. Therefore, further study is needed to properly assess the significance of the finding.

## 5. Conclusion

In conclusion, in this human study, through the comparison of a healthy individual with AECOPD patients at the time of hospital admission and remission, we observed a dramatic expansion of a novel monocyte population, A Mo, in the peripheral blood of AECOPD patients. More importantly, we found the percentage of the A Mo population at admission correlated with hospital stay and disease duration of AECOPD patients. The results suggest that A Mo level may potentially be a biomarker in disease diagnosis and short-term outcome prediction in AECOPD patients. In addition to a much larger size and multicenter study to confirm the finding, further studies should focus on the signature and biological function of A Mo in patients, as well as the relationship of A Mo with disease progression and long-term outcome.

## Figures and Tables

**Table 1:** Study population.

	Healthy	Admission	Remission
Age (yr)	56.23 ± 8.60	74.83 ± 8.92	72.35+ 7.98
Gender (M/F)	8/8 (16)	13/9 (22)	9/8 (17)
FEV1/FVC (%)	80.39 ± 7.50	52.10 ± 12.45^∗∗∗^	
FEV1/pred (%)	90.57 ± 7.01	49.53 ± 11.63^∗∗∗^	
WBC (×10^9^/L)	5.48 ± 1.50	9.60 ± 3.58^∗∗^	8.10 ± 2.01^∗^
Neutrophil (×10^9^/L)	4.51 ± 1.07	7.46 ± 3.27^∗∗^	5.77 ± 2.14^∗∗^
Monocyte (×10^9^/L)	0.33 ± 0.10	0.64 ± 0.30^∗^	0.59 ± 0.21^∗^
Lymphocyte (×10^9^/L)	1.97 ± 0.73	1.38 ± 0.95	1.80 ± 0.51
Neutrophil (%)	51.10 ± 6.30	76.46 ± 14.44^∗∗∗^	68.00 ± 9.94^∗∗∗^^,▲^
Monocyte (%)	5.54 ± 1.01	6.57 ± 3.39	7.61 ± 2.17
Lymphocyte (%)	32.14 ± 5.38	15.92 ± 10.93^∗∗∗^	21.65 ± 8.19^∗∗^^,▲^
CRP (mg/L)	<8.3	39.93 ± 28.89	9.94 ± 7.91^▲▲^
IL-6 (pg/mL)	3.76 ± 1.21	14.24 ± 11.07^∗∗^	11.75 ± 9.79^∗^
IL-8 (pg/mL)	25.43 ± 4.75	77.12 ± 37.96^∗∗∗^	70.08 ± 33.44^∗∗^

M: male; F: female; FEV1/pred: forced expiration volume in one second % predicted FEV1; FEV1/FVC: forced expiratory volume in one second/forced vital capacity; CRP: C-reactive protein; WBC: white blood cells. Data are expressed as the mean ± SD. ^∗^*P* < 0.05, ^∗∗^*P* < 0.01, and ^∗∗∗^*P* < 0.001 versus healthy. ^▲^*P* < 0.05 and ^▲▲^*P* < 0.01 versus AECOPD.

**Table 2:** Surface marker expression of A Mo and T Mo from AECOPD patients and controls.

	%					MFI				
	Healthy	AECOPD		Remission		Healthy	AECOPD		Remission	
	T Mo	T Mo	A Mo	T Mo	A Mo	T Mo	T Mo	A Mo	T Mo	A Mo
CD14	92.79 ± 2.09	97.47 ± 1.45^∗∗∗^	96.66 ± 2.06^∗∗∗^	98.28 ± 0.81^∗∗∗^	96.88 ± 2.04^∗∗∗^	607.00 ± 84.73	441.68 ± 115.60^∗∗^	551.59 ± 149.01	441.41 ± 93.74^∗∗^	521.47 ± 120.94
CD16	13.84 ± 4.83	13.14 ± 11.36	18.76 ± 12.62	8.43 ± 7.14	16.10 ± 9.80	8.21 ± 4.16	8.81 ± 5.07	12.74 ± 7.07^∗∗^	6.41 ± 2.06	10.53 ± 4.49
HLA-DR	83.69 ± 8.95	49.27 ± 12.65^∗∗∗^	66.42 ± 17.92^∗∗∗^	54.05 ± 25.26^∗∗∗^	69.83 ± 22.82^∗∗^	53.71 ± 22.13	28.82 ± 10.25^∗∗∗^	53.44 ± 29.25	39.73 ± 25.32	65.46 ± 39.66
ICAM-1	23.84 ± 4.65	25.14 ± 17.52	33.19 ± 19.44^∗^	26.75 ± 12.16^∗∗^	36.84 ± 12.83^∗∗∗^	21.53 ± 2.89	28.20 ± 8.51^∗^	34.59 ± 12.34^∗∗∗^	31.53 ± 6.92^∗∗∗^	39.59 ± 8.63^∗∗∗^
CCR2	94.69 ± 1.40	96.19 ± 2.67	95.27 ± 3.35^∗^	97.08 ± 2.10	95.30 ± 4.18	252.63 ± 32.97	292.00 ± 75.13	327.68 ± 96.90^∗∗^	307.47 ± 76.62	339.41 ± 81.98^∗∗^

Data are expressed as the mean ± SD, and Student's *t*-test and a nonparametric Newman-Keuls test were used for comparisons between groups. ^∗^*P* < 0.05, ^∗∗^*P* < 0.01, and ^∗∗∗^*P* < 0.001 versus healthy.
